# Supplementary figures and images for: RAB23 loss-of-function mutation causes context-dependent ciliopathy in Carpenter syndrome
Source: PLoS Genet. 2025 Aug 18;21(8):e1011611. doi: 10.1371/journal.pgen.1011611 (PMC12407540; doi:10.1371/journal.pgen.1011611)

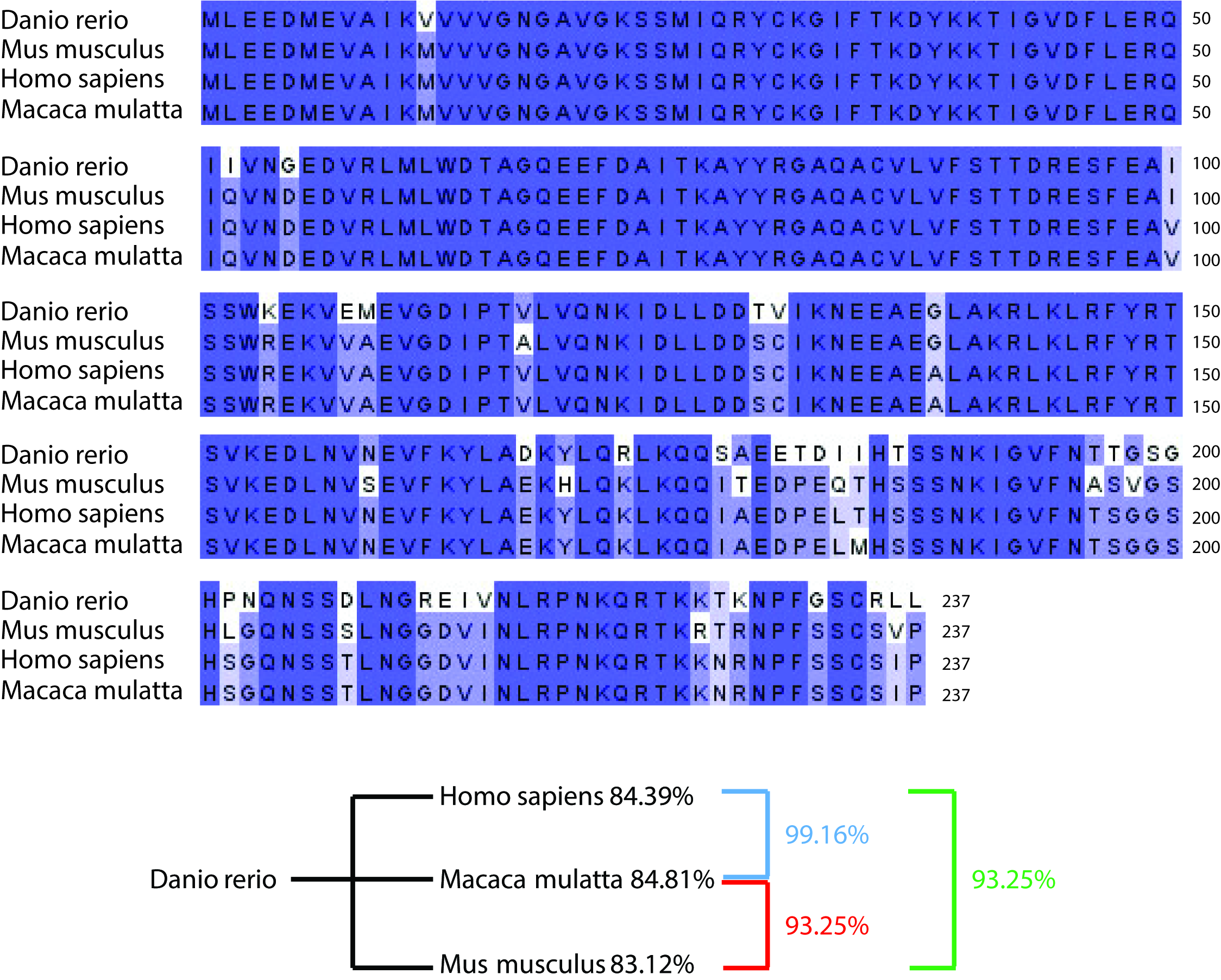

Supplement: S1 Fig — Multiple protein sequence alignment of RAB23 in zebrafish (Danio rerio), mouse (Mus musculus), rhesus macaque (Macaca mulatta) and humans (Homo sapiens). The sequences highlighted in dark blue depict the identical amino acid regions. Danio rerio shares 84.39% identity with human RAB23. Mus musculus shares 93.25% identity with human. (TIF) [file pgen.1011611.s001.tif]

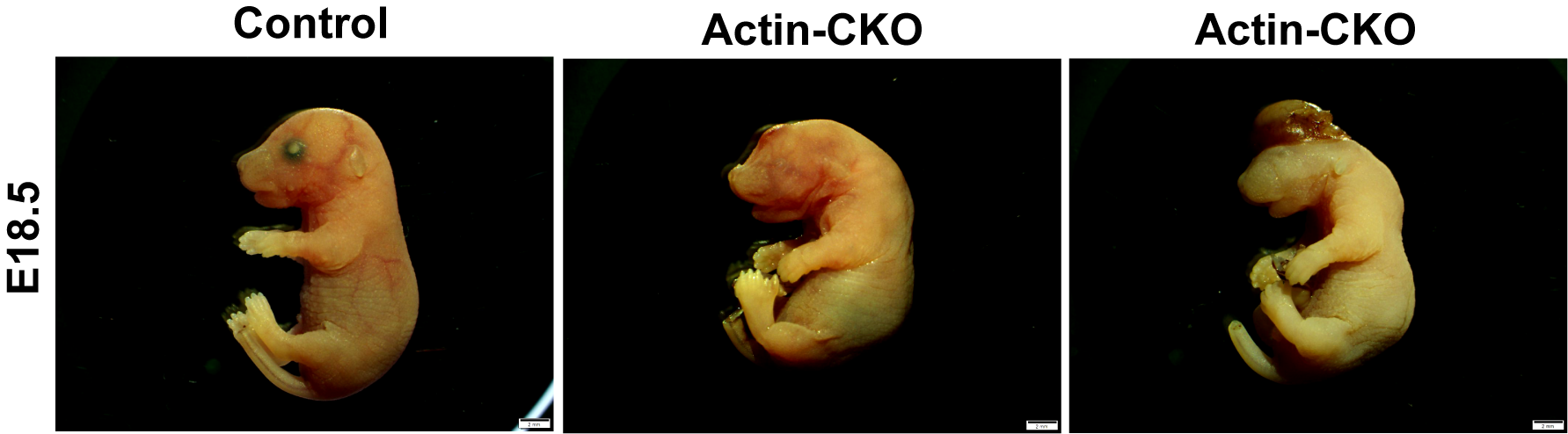

Supplement: S2 Fig — Representative images show morphological appearance of control and actin-CKO mutant mice at E18.5, illustrating actin-CKO mutant mice that display relatively severe brain deformity. (TIF) [file pgen.1011611.s002.tif]

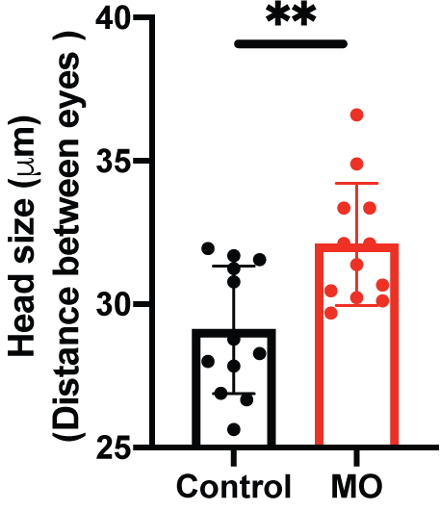

Supplement: S3 Fig — Graph representing quantitative head size measurement between control and rab23 morphant at 72 hpf. The head size was determined by measuring the distance between the eyes in the dorsal view images. The average head size of morphants was slightly yet significantly larger than the control group. n = 12 for each group ** P value ≤ 0.01 Unpaired Student’s t-test. Error bars depict S.D. (TIF) [file pgen.1011611.s003.tif]

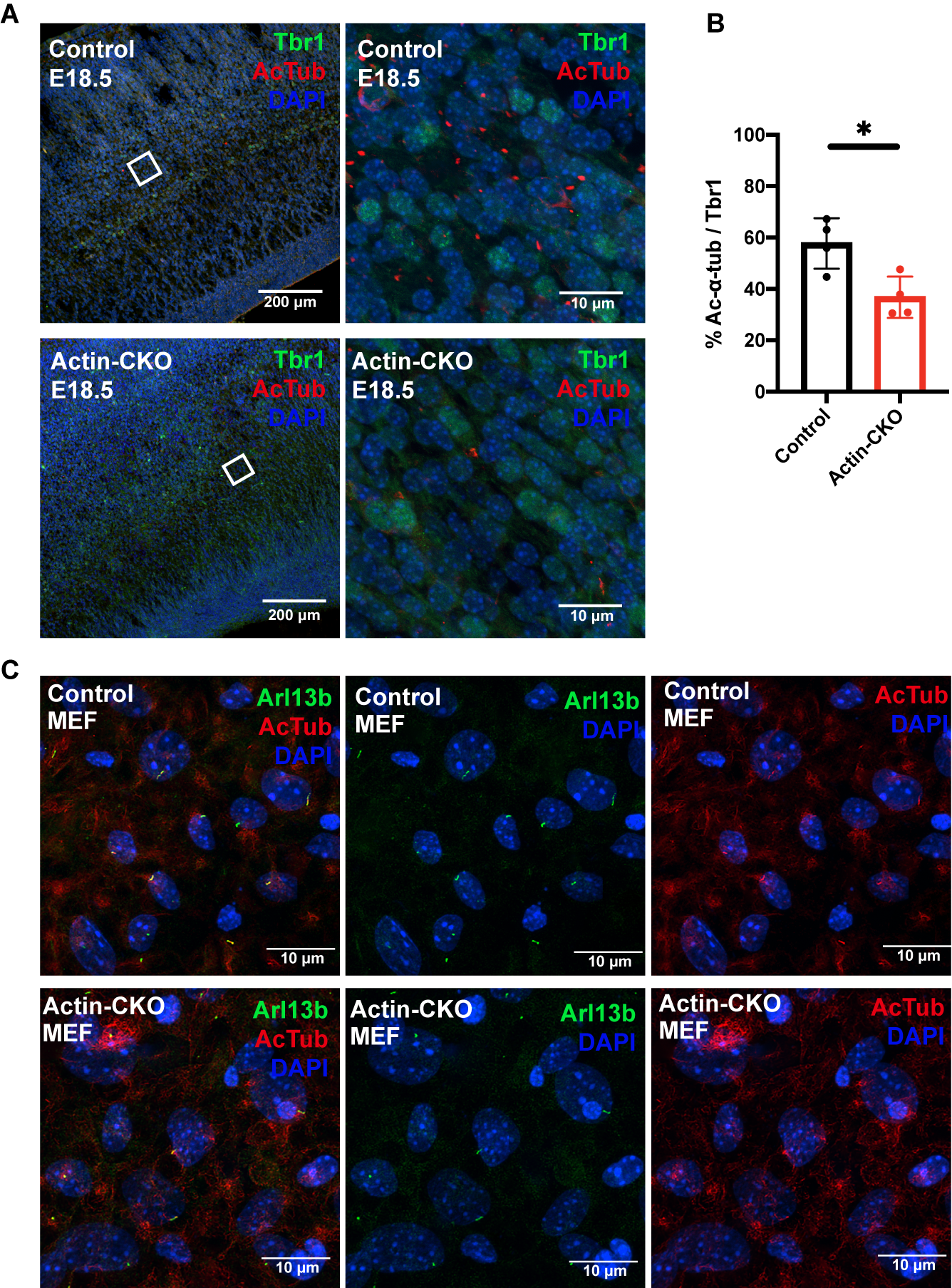

Supplement: S4 Fig — (A-B) Representative immunohistochemistry images (low power on left, magnification of boxed region on right) and (B) graph depicting quantification of the proportion of Acetylated-α-tubulin+ primary cilia against Tbr1+ (green) neocortical layer VI neurons in the neocortex at E18.5. A significant two-fold reduction in the number of primary cilia is observed in the cerebral cortex of actin-CKO mouse embryos. Each dot represents the average percentage count across three brain sections per animal. n = 4 per group. * P value ≤ 0.05, Unpaired Student’s t-test. (C) Representative immunocytochemistry images depicting the Arl13b+ and Acetylated-α-tubulin+ primary cilia in the mouse embryonic fibroblasts cultured from E13.5 control and actin-CKO respectively. (TIF) [file pgen.1011611.s004.tif]

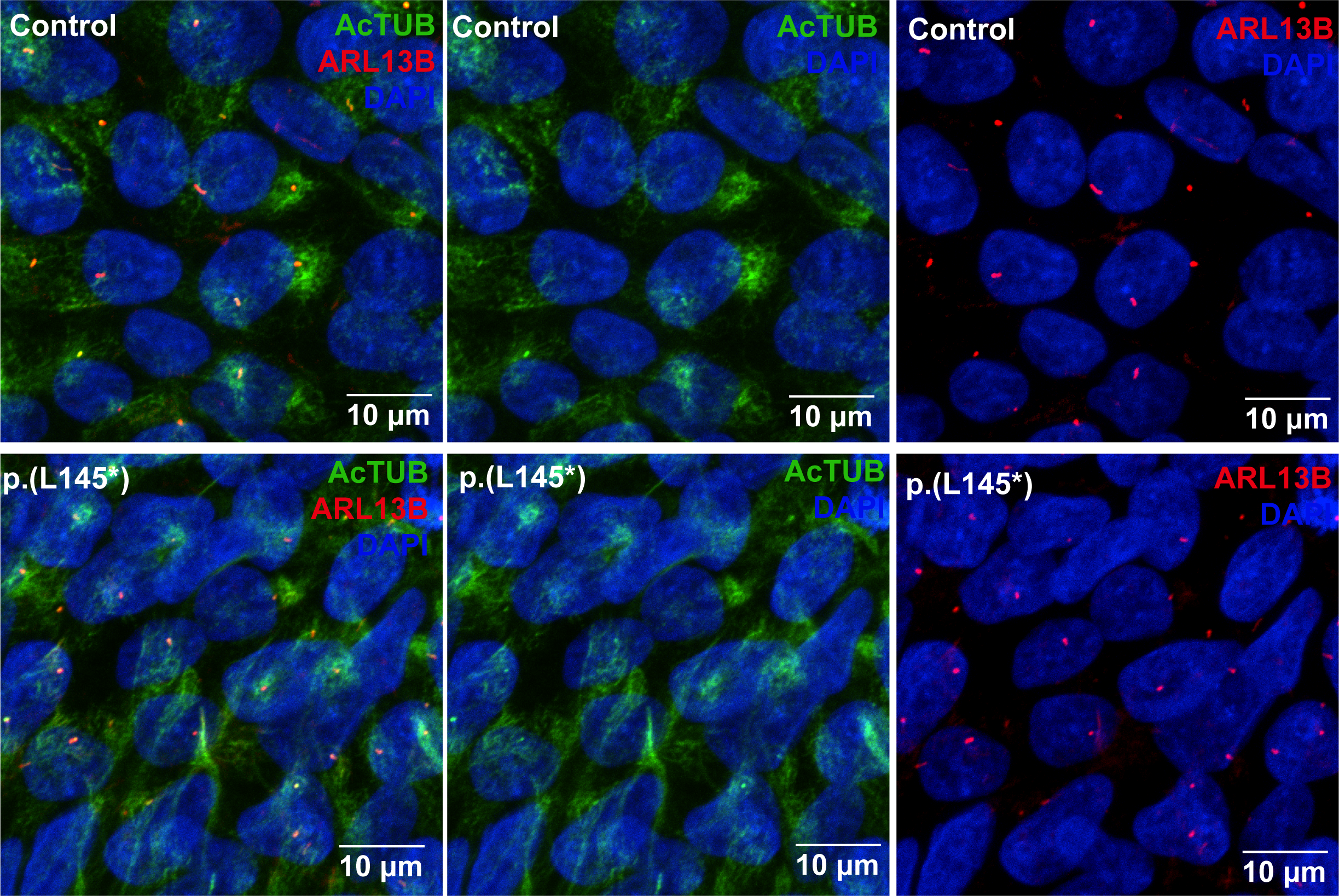

Supplement: S5 Fig — Representative immunocytochemistry images depicting the primary cilia of healthy adult and Carpenter syndrome patient iPSCs co-immunostained for ARL13B and ACETYLATED-α-TUBULIN respectively. (TIF) [file pgen.1011611.s005.tif]
